# Protein overproduction alters exosome secretion in Chinese hamster ovary cells

**DOI:** 10.1007/s00216-023-04725-4

**Published:** 2023-05-09

**Authors:** Aleksandra Steć, Monika Targońska, Edyta Karkosińska, Monika Słowik, Agata Płoska, Leszek Kalinowski, Bartosz Wielgomas, Krzysztof Waleron, Jacek Jasiecki, Szymon Dziomba

**Affiliations:** 1 Department of Toxicology, Faculty of Pharmacy, Medical University of Gdansk, 107 Hallera Street, 80-416 Gdansk, Poland; 2 Department of Biology and Medical Genetics, Medical University of Gdańsk, 1 Dębinki Street, 80-211 Gdańsk, Poland; 3 Department of Medical Laboratory Diagnostics—Fahrenheit Biobank BBMRI.pl, Faculty of Pharmacy, Medical University of Gdansk, 7 Debinki Street, 80-211 Gdansk, Poland; 4 BioTechMed Centre, Department of Mechanics of Materials and Structures, Gdansk University of Technology, 11/12 Narutowicza Street, 80-233 Gdansk, Poland; 5 Department of Pharmaceutical Microbiology, Faculty of Pharmacy, Medical University of Gdansk, 107 Hallera Street, 80-416 Gdansk, Poland

**Keywords:** Capillary electrophoresis, Chinese hamster ovary cells, Exosomes, Extracellular vesicles

## Abstract

Despite the abundance of available cell lines, nearly 70% of all recombinant therapeutic proteins today are produced in Chinese hamster ovary (CHO) cells. The impact of protein overproduction on the secretion of exosomes by CHO cells has been investigated here. Increased secretion of extracellular vesicles (EVs) by protein overexpressing CHO cells was demonstrated with protein content assay, nanoparticle tracking analysis, and capillary electrophoresis. Our results revealed that a protein overproduction might induce EVs secretion, which might be accompanied by the sequestration and loading of overexpressed proteins into the exosomes. These findings are of vital importance for the manufacturing of therapeutics in CHO expression systems due to the risk of product loss during downstream processing of culture medium as well as the application of exosomes as nanocarriers of therapeutic proteins. The study indicates also the importance of culturing process control.

**Supplementary Information:**

The online version contains supplementary material available at 10.1007/s00216-023-04725-4.

## Introduction

Chinese hamster ovary (CHO) cells are the most widely used as an expression system for recombinant therapeutic protein (RTP) production, and nearly 70% of RTPs are currently generated by CHO cells, especially monoclonal antibodies (mAbs). Almost all (close to 90%) commercially available mAbs were produced in CHO cells, corresponding to a market value of $107 billion [1, 2]. The popularity of the usage of CHO cells for RTP production is based on their unique advantages, i.e., a variety of post-translational modifications of the produced proteins in CHO are highly similar to those in human cells; well-proven genetic tools are available to optimize CHO cells; low risk of propagation of human viruses due to the hamster origin. Moreover, CHO cells are accessible to culture and grow well in suspension and as an adherent culture with a shorter generation time than human cells. Their tolerance to oxygen, carbon dioxide levels, temperature, and pH variations makes them suitable cells for large-scale culture. Furthermore, CHO can be cultivated in animal-free, protein-free, serum-free medium (SFM), decreasing the risk of contamination by animal-origin viruses or proteins of biopharmaceutical products. High recombinant protein yields and specific productivity cause CHO cells to be used to produce about 50 biotherapeutics already approved in the USA and EU [3–5].

Extracellular vesicles (EVs) are small lipid bilayer extracellular particles derived from cytoplasmic or endosomal membranes and are produced and secreted by a wide variety of different cells, including CHO cells. EVs carry a cargo of important cellular biomolecules, including proteins, nucleic acids, lipids, metabolites, and organelles. Cells use EVs to mediate intercellular communications and send stress signals and small RNAs to alter the expression and expel toxic accumulated products [6, 7].

The EVs, secreted by CHO cells, have recently been characterized. A single CHO cell was estimated to produce up to about 200 EVs (nano- and microvesicles) per day [8]. The analysis showed that exosomes produced by CHO cells are composed of more than 1300 proteins and hundreds of non-coding RNAs [9]. An intense exchange of cellular material between cells during culturing and adjustment of the EVs’ composition depending on the cell growth phase indicate their vital role in cell-to-cell communication which seemed to be underestimated in bioprocessing. Han and Rhee reported inhibition of CHO cell apoptosis by CHO-derived EVs [10]. The authors indicated that EVs might be used as growth promotors to increase the viability of cells and bioprocess productivity without safety concerns. The concept was adopted by Takagi et al. [11]*.* The group proposed repeated batch culture of CHO cells with the reuse of an EVs-rich fraction of culturing medium. The vesicles in reused fraction were <220 nm and were CD81 positive. The utility of CHO cells’ exosomes has recently been demonstrated by Seras-Franzoso and coworkers [12]. The authors used CHO DG44 and HEK293 cell lines as expression systems for the production of enzymes related to lysosomal storage disorders (LSDs). Both investigated enzymes (alpha-galactosidase A and N-sulfoglucosamine sulfohydrolase) were found to be secreted through EVs. The isolated vesicles were parenterally administered to animal models of LSDs, delivering therapeutic effects and advantageous pharmacokinetics as compared to conventional therapy.

The aim of the presented work was to investigate the impact of transfection on EVs production by CHO cells. It was verified whether the type of overproduced protein influences the intensity of EVs secretion as well as the physicochemical properties of vesicles. To verify these hypotheses, plasmids encoding butyrylcholinesterase (BChE) and β-galactosidase (β-Gal) were used which enabled us to verify if the secretion of overproduced enzymes is EVs-dependent. A side goal of the study was to demonstrate the applicability of the capillary electrophoresis technique (CE) to monitor the EVs isolation process from eukaryotic cell cultures.

## Materials and methods

### Materials

BIS-Tris propane (BTP), glycine (Gly), sodium dodecyl sulfate (SDS), phosphate-buffered saline (PBS), bovine serum albumin (BSA), sodium chloride, Tween 20, and ***tris*** (hydroxymethyl) aminomethane (Tris) were obtained from Merck (Darmstadt, Germany). Sodium hydroxide was purchased from Avantor (Gliwice, Poland). All chemicals were of analytical grade. Deionized water was obtained with the Basic 5 water purification system (Hydrolab, Wislina, Poland).

ExpiCHO™ Expression System was obtained from Thermo Fisher Scientific (Waltham, MA, USA).

The 4 mg mL^−1^ stock solution of 2-nitrophenyl β-d-galactopyranoside (ONPG; Merck) was prepared in 0.1 M phosphate buffer (pH 6.8; Merck). To obtain the ONPG reagent solution, the ONPG stock solution was mixed with 0.1 M MgCl_2_ (Merck) aqueous solution and 0.1 M phosphate buffer solution in the volumetric ratio of 66:3:201, respectively.

The SDS-PAGE buffer (25 mM Tris, 192 mM glycine, 0.1% m/v SDS), transfer buffer (25 mM Tris, 192 mM glycine), and TBST buffer (Tris Buffered Saline; 20 mM Tris 7.5 pH, 150 mM NaCl, 0.1% v/v Tween 20) were prepared in deionized water and used in western blot experiments.

### Expression vector

pCMV Sport-βgal vector encoding β-Gal was purchased from Thermo Fisher Scientific. pTracerBCHE vector expressing the C-terminal truncated form of BChE was constructed by using cloning techniques. An insert encoding BChE was amplified by polymerase chain reaction (PCR) method using template EX-A0103-M51 (GeneCopoeia, Inc) and specific primers: forward NBCHECHO-5′-ATAGATATCAATATGCATAGCAAAGTCACAATC and revers CCHOBCHEHIS-5′-ATAGATATCTTAGTGATGGTGATGGTGATGGACTTTTGGAAAAAATGATGTCCAGAATCG. The insert was cloned into the EcoRV restriction site of the expression vector pTracer-CMV/Bsd (Thermo Fisher Scientific) and introduced into *E. coli* Stellar™ (Takara Bio, Japan).

### Cells culturing and transfection

Chinese hamster ovary (CHO) cells were maintained in ExpiCHO™ Expression Medium (Thermo Fisher Scientific, USA) at 37°C with a humidified atmosphere of 8% v/v CO_2_ on an orbital shaker at 125 rpm. Cells were transfected with appropriate plasmid DNA after reaching 6 × 10^6^ cells/mL cells’ density. The plasmid DNA and ExpiFectamine™ CHO Reagent were diluted separately with OptiPRO™ medium (Thermo Fisher Scientific, Lithuania) according to the manufacturer’s instructions, connected into one mixture, and incubated for 5 min at room temperature (RT). After this time, ExpiFectamine™ CHO/plasmid complexes were added to the cells. The cells were cultured according to the standard protocol for 8 days. Subsequently, the cultures were centrifuged at 8000 g, 4°C, for 10 min, and the supernatants were kept for the next analyses.

### Isolation of vesicles

The culturing media was centrifuged for 30 min at 3000 g at the temperature of 4 °C. 10 mL of collected supernatant was ultrafiltrated down to 0.5 mL with Vivaspin 20 concentrator (100 kDa MWCO, PES, Sartorius) according to the vendor’s recommendations. The retentate was fractionated into 25 fractions, 0.5 mL each, with qEVoriginal 35 nm Gen 2 (IZON) SEC column. The elution was conducted with PBS solution. The obtained fractions were stored at 4 °C.

### Protein assay

Total protein content assay was conducted with Pierce BCA Protein Assay Kit (Thermo Fisher Scientific, Waltham, MA, USA) according to vendors’ recommendations. Bovine serum albumin (BSA) was used for calibration. The samples and standards were mixed with 6% m/m SDS solution in a 9 to 1 volumetric ratio. The measurements were performed in 96-well plates using an Infinite M200 plate reader (Tecan, Mannedorf, Switzerland).

### Nanoparticles tracking analysis (NTA)

NTA analysis was performed with the Nanosight NS300 instrument (Malvern Instruments, UK) and controlled with the NTA software (version 3.2 Dev Build 3.2.16, Malvern Instruments, UK). Samples were diluted in PBS to obtain around 40–100 particles per frame. A 305-nm laser and an sCMOS camera (camera level = 15–16; detection threshold = 5; focus = 180–220; flow rate = 100) were used to track particles. Each sample was analyzed twice. The single analysis included 5 films (60 s each).

### Capillary electrophoresis (CE)

The CE experiments were performed with PACE MDQ plus system (Sciex, Framingham, MA, USA) equipped with a photodiode array detector and controlled with 32 Karat software (version 10.2, Sciex). Separation was conducted in uncoated silica capillaries (50 μm i.d. × 30.2 cm of total length; Polymicro Technologies, West Yorkshire, UK). The background electrolyte was composed of 50 mM BTP and 75 mM Gly (pH 9.5). The sample was injected hydrodynamically (5 s, 3.45 kPa). The electrophoresis was carried out at 10 kV (25 °C) and the separation process was monitored at 200 and 230 nm. Capillary conditioning was described elsewhere [13].

### Transmission electron microscopy (TEM)

TEM analysis was performed with Tecnai G2 T12 Spirit BioTwin microscope (FEI Company, Hillsboro, OR, USA). Before the analysis, the isolates (5 μL) were deposed on the formvar support on a copper mesh (200 mesh, Agar Scientific, Stansted, UK), contrasted with a 1% m/v uranyl acetate, and left for drying.

### β-Galactosidase activity assay

270 μL of ONPG reagent solution was mixed with 30 μL of a sample. The absorbance was measured with an Infinite M200 plate reader (Tecan) at 420 nm wavelength in a time interval of 1 min. The β-galactosidase activity was determined as an increment of absorbance in time.

### Measurement of BChE activity by Ellman’s assay

BChE activity of each SEC fraction was determined spectrophotometrically by modified Ellman’s method [14] using BTC (S-butyrylthiocholine iodide) as a substrate. The assay was performed in 96-well microtiter plates in a final reaction volume of 200 μL of 100 mM phosphate buffer (pH 7.4) with a final concentration of 0.5 mM DTNB (5,5′-Dithiobis(2-nitrobenzoic acid) and 5 mM BTC as described previously [15]. The absorbance was monitored at 412 nm by repeated measurements at 1-min intervals for 10 min by a microplate reader spectrophotometer (Tecan Infinite M200 Pro, Tecan Group Ltd., Männedorf, Switzerland) at 25 °C.

### Western blot

The SEC fractions were concentrated with Amicon ® Ultra 10K (Merck) concentrator. The total protein concentration of samples was measured with a DC protein assay (Bio-Rad, Hercules, CA, USA). On each lane of the 12% m/v SDS-PAGE gel, 30 mg of total protein amount from certain fractions was loaded. The proteins were separated at 100V for 90 min. Next, the proteins were blotted onto a PVDF membrane by using a wet transfer system at 100 V for 1 h. The membrane was blocked with TBST buffer with 3% m/v skim milk for 1 h at 4°C. The proteins were detected using the primary rabbit monoclonal antibodies: anti-Hsp70, anti-TSG101, anti-CD63 (Abcam, Trumpington, UK), and the secondary antibodies anti-rabbit IgG horseradish peroxidase conjugate (Bio-Rad, USA). The signals were measured in the ChemiDoc Touch Imaging system (Bio-Rad, USA) with a Clarity Western ECL Substrate chemiluminescence kit (Bio-Rad, USA).

## Results

### Isolation and characterization of EVs

**Fig. 1:**
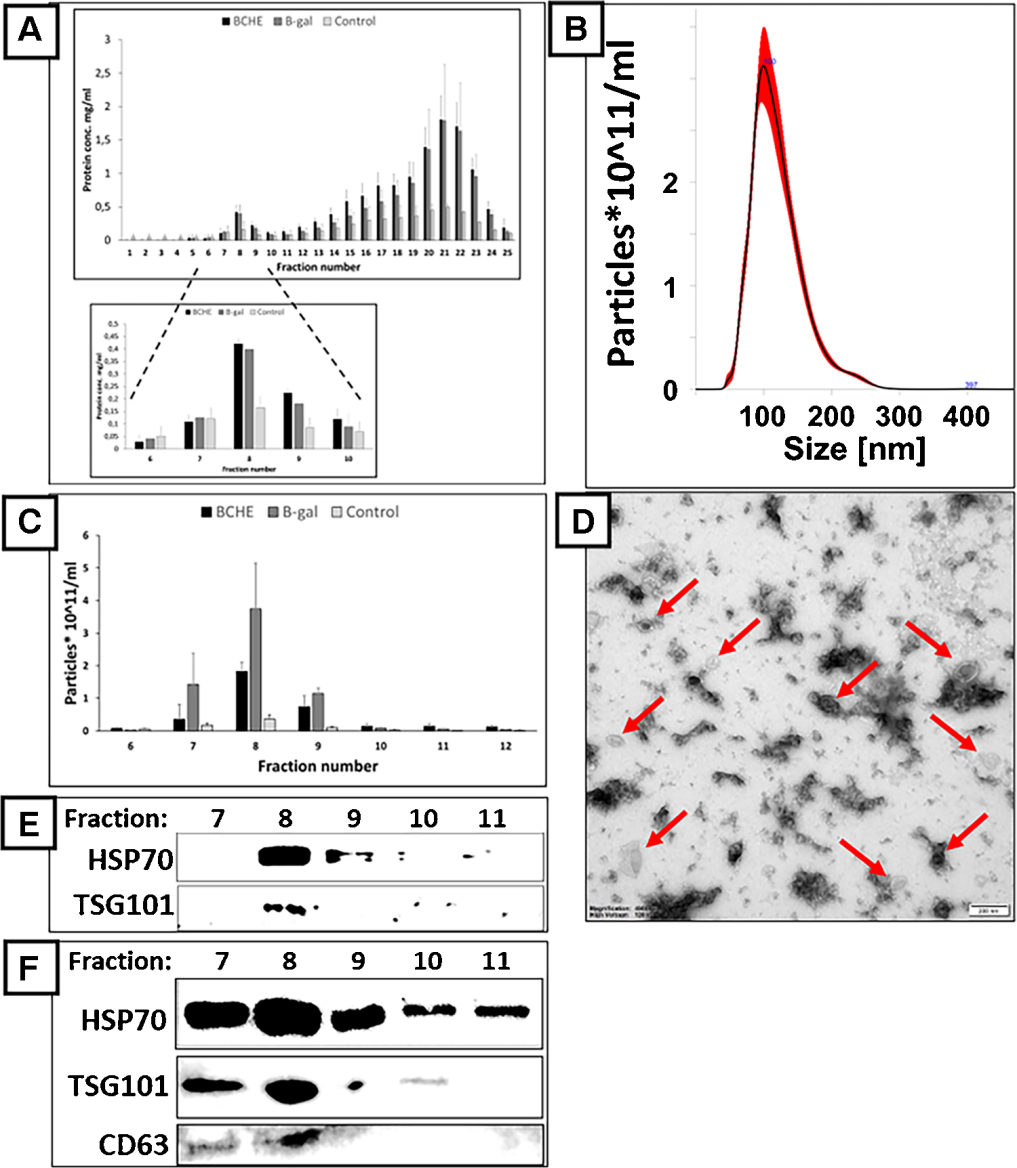
The analysis of fractions obtained with SEC from the culturing medium of CHO cells transfected with vectors overexpressing BChE (black bars); β-Gal (grqy bars) and untransfected control; (light gray bars). (**A**) Total protein concentration assay of SEC fractions (at least four measurements in two independent experiments were performed per bar; the results were shown as an average ± standard deviation). The bottom graph shows the enlarged view of the 6^th^–12^th^ fractions. (**B**) Particle size distribution of 8^th^ fraction obtained from β-Gal-overproducing cell cultures measured with NTA. (**C**) Particle concentration in selected fractions was measured with NTA (at least two measurements in two independent experiments were performed per bar; the results were shown as an average ± standard deviation). (**D**) TEM image of 8^th^ fraction obtained from β-Gal-overproducing cells cultures (scale bar = 200 nm). (E, F) Exosome protein markers, HSP 70, TSG101, CD63 detection with western blotting in SEC fractions from the culturing medium of BChE- (**E**) and β-Gal-overproducing (**F**) cells

The isolation of EVs was performed according to the procedure described in the “Isolation of vesicles” section. The culturing medium was centrifuged, the supernatant was preconcentrated, and the retentate was separated with SEC columns obtained from IZON. According to column specification, fractions 7^th^–9^th^ were expected to contain vesicles. Indeed, the BCA (bicinchoninic acid) protein assay revealed the presence of proteins in fractions 7^th^–9^th^ (Fig. 1A). These fractions were also rich in particles (Fig. 1B and C) with a modal size of about 100 nm and particle size distribution ranging from 40 to 270 nm (Fig. 1B and  S1). The size distribution of EVs in all analyzed isolates was found similar (Figs. 1B and S1). TEM analysis confirmed the presence of vesicles in all analyzed fractions (8^th^ fractions from each sample type; Figs. 1D and S1). No morphological differences between samples were observed. WB analysis has finally confirmed the presence of exosomal markers HSP70, TSG101, and CD63 whose concentration peaked in fractions 7^th^–9^th^ (Fig. 1E and F). Attention should be paid that the culturing medium was serum-free (“Cells culturing and transfection” section). Thus, we have not screened the fractions with WB for negative markers presence.

Significant amounts of proteins were detected in fractions from the 10^th^ to 25^th^ (Fig. 1A) but these fractions were devoid of nanoparticles (Fig. 1C). This observation is also in line with column characteristics as later eluting fractions are assumed to contain soluble proteins and other molecular components of the samples.

**Fig. 2:**
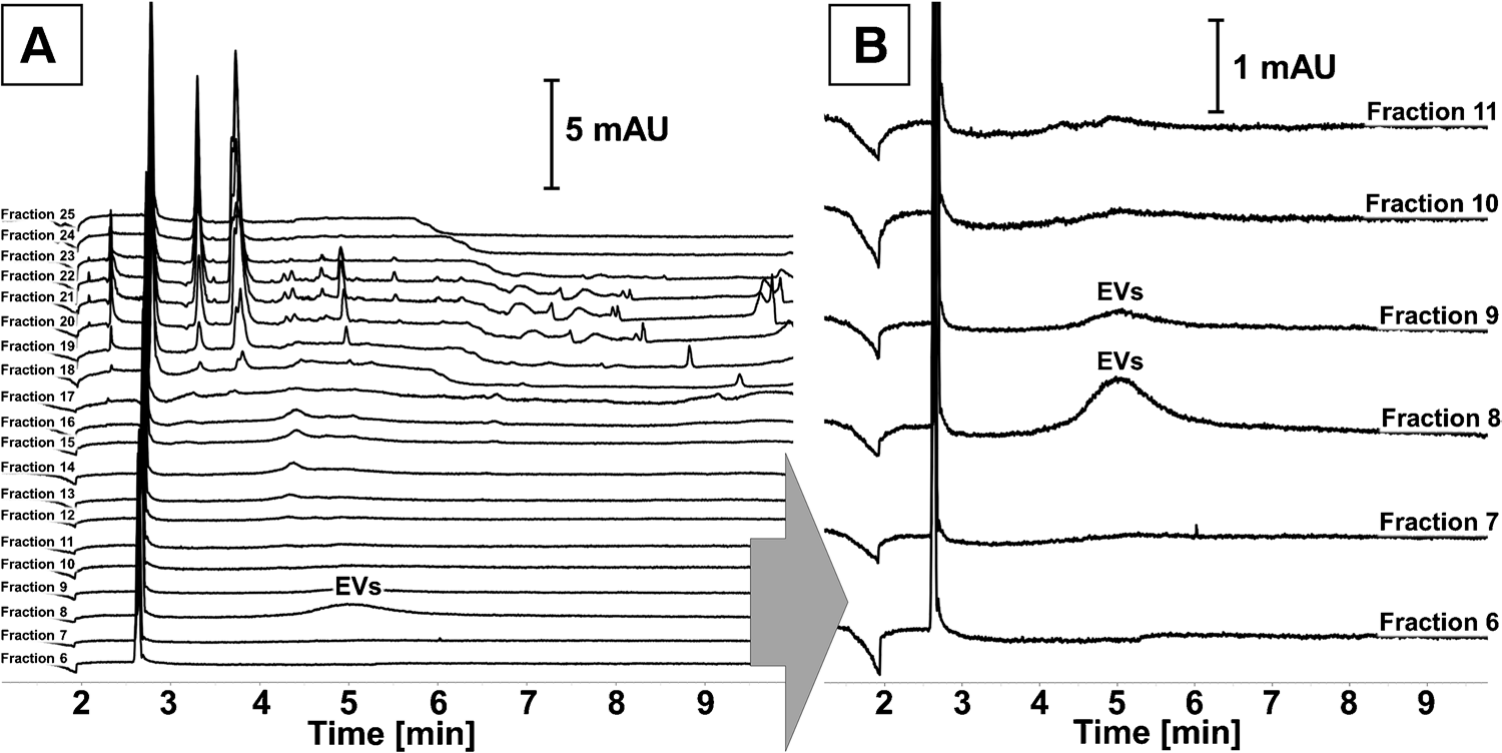
The CE analysis of fractions obtained with SEC from the culturing media of CHO cells overexpressing β-Gal. Figure B shows an enlarged view of A. Experimental conditions were provided in the “Capillary electrophoresis (CE)” section

Similar conclusions were drawn from CE analyses. Characteristic low-efficient signals (*N* < 20,000 plates/m) were detected in fractions 8^th^ and 9^th^ and were not present in later eluting fractions (Fig. 2A and B) [13, 16–19]. Such signals are typically generated by EVs in CE. Numerous signals, which might be assigned to proteomic and low-molecular sample matrix components, were detected in later eluting fractions (especially fractions 18^th^–25^th^; Fig. 2A). The CE analysis of later eluting fractions might provide information on the efficiency and yield of products secreted by CHO cells to the culturing medium. However, it was out of the scope of the presented work. Nevertheless, the sensitivity of CE with UV detection was sufficient to detect EVs in isolates obtained from culturing media of CHO cells transfected with genes encoding BChE and β-Gal.

Both proteins and particles were found in fractions from 7^th^ to 9^th^ in all analyzed samples which indicates that vesicles were secreted by transfected and untransfected CHO cells. All three techniques (BCA, NTA, and CE) showed that the overproduction of β-Gal and BChE proteins increased the secretion of EVs by the CHO cells (Figs. 1 and S2). Increased secretion of EVs by these cells was accompanied by increased secretion of soluble proteins and other molecular components of the sample (Fig. 1A).

### EVs cargo

Increased secretion of EVs by transfected CHO cells made us verify whether translated proteins are loaded into vesicles. While the increased secretion of EVs was observed in the case of cells transfected with genes encoding BChE and β-Gal, the enzymatic activity in all obtained SEC fractions was assessed according to the procedures described in the “β-Galactosidase activity assay” and “Measurement of BChE activity by Ellman’s assay” sections.

**Fig. 3:**
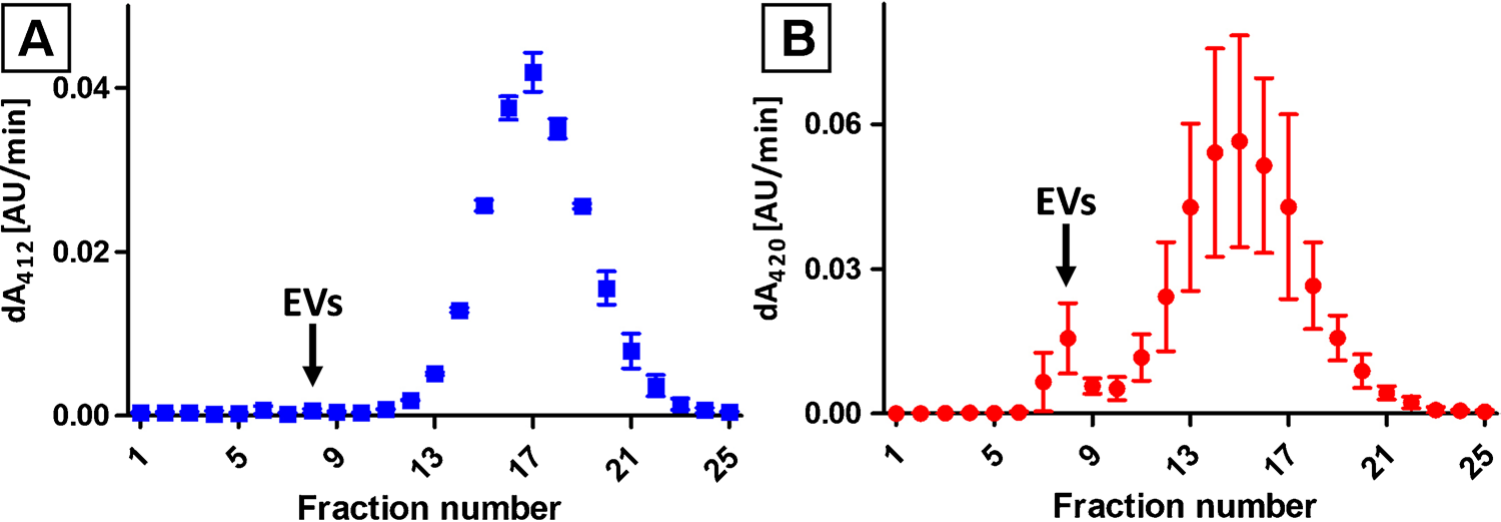
The enzymatic activity of BChE (**A**) and β-Gal (**B**) in certain SEC fractions of media harvested from CHO cell culture transfected with the corresponding protein-expressing vectors. EVs fractions: 7^th^–9^th^, the enzymatic activity of the secreted proteins in fractions: 13^th^–22^nd^. The results were presented as an average ± standard deviation obtained in two independent experiments. Experimental conditions were provided in the “β-Galactosidase activity assay” and “Measurement of BChE activity by Ellman’s assay” sections

The assays showed high enzymatic activity of later eluting fractions (12^th^–23^rd^) obtained from culturing media of cells expressing BChE and cells translating β-Gal (Fig. 3A and B, respectively) which confirmed the effectiveness of the transfection process and the overproduction of the protein. However, cholinesterase activity in EVs-rich fractions (7^th^–9^th^) was found negligible and accounted for <0.5% of summary enzymatic activity measured in all SEC fractions (Fig. 3A). Interestingly, a significant β-Gal activity (7.2 ± 0.7% of total activity measured in all fractions) was found in fractions 7^th^–9^th^. The measured activity correlated with the EVs content in these fractions.

## Discussion

EVs are released by cells to the extracellular environment to mediate intercellular communication. These nanocarriers transport proteins, lipids, nucleic acids, and metabolites from parental cells, reflecting the nature of the donor cell and its physiological state [20].

EVs in the culture are in constant turnover, being secreted and uptaken by cultured cells [1]. EV exchange allows a culture of cells to share protein and RNA that can regulate cellular processes in the whole culture as in individual cells. Recent studies have reported that exosomes regulate many biological processes, including apoptosis, inflammation, and differentiation [10, 21]. Moreover, exosome composition, production, and secretion can be affected by the external stimuli, including stress conditions such as heat shock, oxidative stress, chemotherapy, irradiation, hypoxia, and hypothermia. One of them is a cellular stress response (CSR) that is triggered upon recombinant protein synthesis. The high-level scale recombinant proteins overexpression may impact host cell metabolism, increase energy production, and improve secretory capacity, leading to a rise of reactive oxygen species and promoting cellular and oxidative stress or the unfolded protein response (UPR) [6, 20, 22, 23].

Several studies have reported that exosomal proteins are loaded through various sorting mechanisms. It was reported that the composition of the exosomal cargo and proteins targeting EVs is regulated by their post-translational modifications (PTM) [24]. Multiple mechanisms involved in exosome biogenesis have been identified. In the classical pathway, exosomes are formed within the endosomal system from intraluminal vesicles (ILVs) in late endosomes/multivesicular bodies (MVBs). ILV biogenesis and secretion are driven by the ESCRT proteins machinery with SNARE and other proteins. MVBs are specialized endosomal compartments that sequester proteins, lipids, and potential exosome cargoes delivered from the trans-Golgi network and the cytosol. MVBs with intraluminal vesicles get transported to the plasma membrane, fuse with the cell membrane, and release the internal ILVs into the external environment as secreted exosomes [6, 25].

In the presented study, CHO cells were grown in a serum-free medium which eliminated the risk of isolates contamination with EVs of animal origin. Therefore, isolated EVs were derived from CHO cells. The characterization confirmed that the isolates obtained in the presented study contained mainly exosomes. Most particles detected with NTA featured size in the range of 40–200 nm, which was confirmed with TEM analysis (Figs. 1B and D and S1). Moreover, the abundance of several exosome-specific markers was demonstrated (Fig. 1E and F) [20]. Bigger particles (>200 nm) detected with NTA and TEM suggest that isolates also contained microvesicles (Figs. 1B and D and S1). However, the same data shows that microvesicles were in the minority, and their content was estimated at <5%.

This work presents the overexpression of β-Gal and BChE proteins in CHO cells from the expression vectors. BChE is a member of the hydrolase that catalyzes the hydrolysis of choline and non-choline esters. BChE exhibits broad substrate specificity and is involved in the detoxification of poisons, including organophosphate nerve agents and pesticides, and the metabolism of drugs such as cocaine and heroin. BChE functions as a natural scavenger, like a sponge, and traps toxic compounds present in the plasma, either hydrolyzing or binding them permanently, preventing them from reaching AChE present in peripheral nerve connections or the central nervous system, before they cause neurological damage. The main source of the purified BChE is human plasma (Cohn fraction IV), but the amount of protein that can be obtained in this way is insufficient for the needs. Various attempts have been made to develop more efficient BChE production technologies based on genetic engineering technologies. Attempts have been made to overproduce BChE in various expression systems in vitro or as transgenic plants and animals [26]. In 1997, a recombinant BChE was obtained in the CHO cell line [27]. Here, the BChE protein and its overexpression in CHO cells have been studied because the protein overproduction is at high yield, the post-translational modifications are well characterized, and the method of enzyme detection is very simple and sensitive [14]. Even a small amount of the enzyme could be detected in exosomes. β-Gal expressing vector, pCMV·SPORT-βgal, is a positive control for monitoring expression in eukaryotic cells. The plasmid contains the reporter gene β-galactosidase (β-gal) from *E. coli* cloned into plasmid pCMV·SPORT1.

The results indicate that protein overexpression can trigger CSR and increase exosome production. Interestingly, during BChE overproduction, exosome secretion is enhanced, but only trace BChE activity was observed in the exosome fractions. These findings are in line with the report of Liao and coworkers [28]. The authors demonstrated no correlation between acetylocholinesterase activity in isolates obtained from various cell lines and particle count. It has also been shown that the enzymatic activity was predominantly associated with soluble fraction obtained during centrifugation. Noteworthy is the fact that other groups reported both cholinesterase activity and the detection of scant enzymes in EVs isolates obtained from numerous cell lines [29, 30]. However, it might be assigned to the contamination of the isolates with soluble enzymes or co-isolation of the cholinesterase as a part of EVs corona [31]. For instance, BChE was detected in isolates obtained from only two cell lines among sixty taken into assay [30].

β-Gal overproduction in CHO cells also resulted in increased secretion of EVs. Contrary to BChE overproduction, the isolates obtained from cells overproducing β-Gal featured significant enzymatic activity (Fig. 3B). The secretion of α-galactosidase A (α-Gal) with EVs has already been presented by Seras-Franzoso and co-authors [12]. The similarity of α- and β-Gal implies an analogous secretory pathway, distinct from BChE. Attention should be paid that α- and β-Gal are lysosomal enzymes (also cytosolic). The involvement of lysosomes in MVB formation might explain the sequestration of β-Gal into exosomes which was not observed in the case of BChE overproduction.

It was estimated that 7.2 ± 0.7% of β-Gal, secreted by CHO cells, was carried by EVs. This fact might be utilized for therapeutic protein encapsulation into exosomes [12]. The application of exosomes as nanocarriers might be beneficial for the stability and targeted delivery of incorporated protein. It has been shown that cytokines carried by EVs featured superior stability as compared to free cytokines and released cellular response after vesicle internalization [32]. Genetic engineering methods might also be used for the expression of surface receptors for bioactive compounds bounding [33]. On the other hand, EVs-mediated secretion of therapeutic proteins, expressed in CHO (and probably other) cell lines, should be considered due to the risk of loss during downstream processing of culturing media. The discussed case indicates also the importance of culturing process control. The presented work addresses this need, demonstrating the potential of the CE technique in the monitoring of culturing media composition. Good agreement between BCA, NTA, and CE results of analysis of SEC fractions was observed (Figs. 1 and 2). It should be emphasized that CE was able to provide qualitative and quantitative information on analyzed fractions. As a result, not only the relative content of EVs in certain fractions could be assessed, but also the purity of the isolates. Furthermore, despite it not being investigated in presented work, it might be assumed that the method can be used for simultaneous qualitative and quantitative analysis of proteins (e.g., secreted by the transfected cells).

## Conclusion

Optimization of the production of biological drugs is fundamental in a view of product quality, process yield, and the economical balance of the manufacturing trial. The presented work refers to these issues showing the increased secretion of EVs by the cells employed to overproduction of model enzymes of therapeutic significance. Based on the presented results, the EVs-mediated secretion of produced protein should be verified and recovery of produced protein from vesicles should be considered during downstream processing of culturing medium. Moreover, the encapsulation process might be utilized for targeted therapeutics production, better control of manufactured EVs-based therapeutics potency and improvement of pharmacokinetics of biological drugs. Nevertheless, the secretion of EVs by cultured cells might be monitored using commonly used methods (protein content tests or particle counting techniques) as well as alternative techniques like the presented CE. The latter was shown to not only assess the relative content in certain isolates but also provide information on the purity of the isolates.

## Supplementary Information

ESM 1 (DOCX 1351 kb)
