# Supplementary material for: Protein overproduction alters exosome secretion in Chinese hamster ovary cells
Source: Anal Bioanal Chem. 2023 May 9;415(16):3167–76. doi: 10.1007/s00216-023-04725-4 (PMC10287810; doi:10.1007/s00216-023-04725-4)
Supplement: Supplementary file 1 — (DOCX 1351 kb) [file 216_2023_4725_MOESM1_ESM.docx]

*Supplementary Materials*

**Protein overproduction alters exosome secretion in CHO cells**

Aleksandra Steć^a^*, Monika Targońska^b^*, Edyta Karkosińska^a^, Monika Słowik^a^, Agata Płoska^c^, Leszek Kalinowski^c,d^, Bartosz Wielgomas^a^, Krzysztof Waleron^e^, Jacek Jasiecki^e^**, Szymon Dziomba^a^**

^a^ Department of Toxicology, Faculty of Pharmacy, Medical University of Gdansk, 107 Hallera Street, 80-416 Gdansk, Poland

^b^ Department of Biology and Medical Genetics, Medical University of Gdańsk, 1 Dębinki Street, 80-211 Gdańsk, Poland

^c^ Department of Medical Laboratory Diagnostics—Fahrenheit Biobank BBMRI.pl, Faculty of Pharmacy, Medical University of Gdansk, 7 Debinki Street, 80-211 Gdansk, Poland

^d^ BioTechMed Centre, Department of Mechanics of Materials and Structures, Gdansk University of Technology, 11/12 Narutowicza Street, 80-233 Gdansk, Poland

^e^ Department of Pharmaceutical Microbiology, Faculty of Pharmacy, Medical University of Gdansk, 107 Hallera Street, 80-416 Gdansk, Poland

*These authors contributed equally.

**Corresponding authors:

jacek.jasiecki@gumed.edu.pl

szymon.dziomba@gumed.edu.pl

***
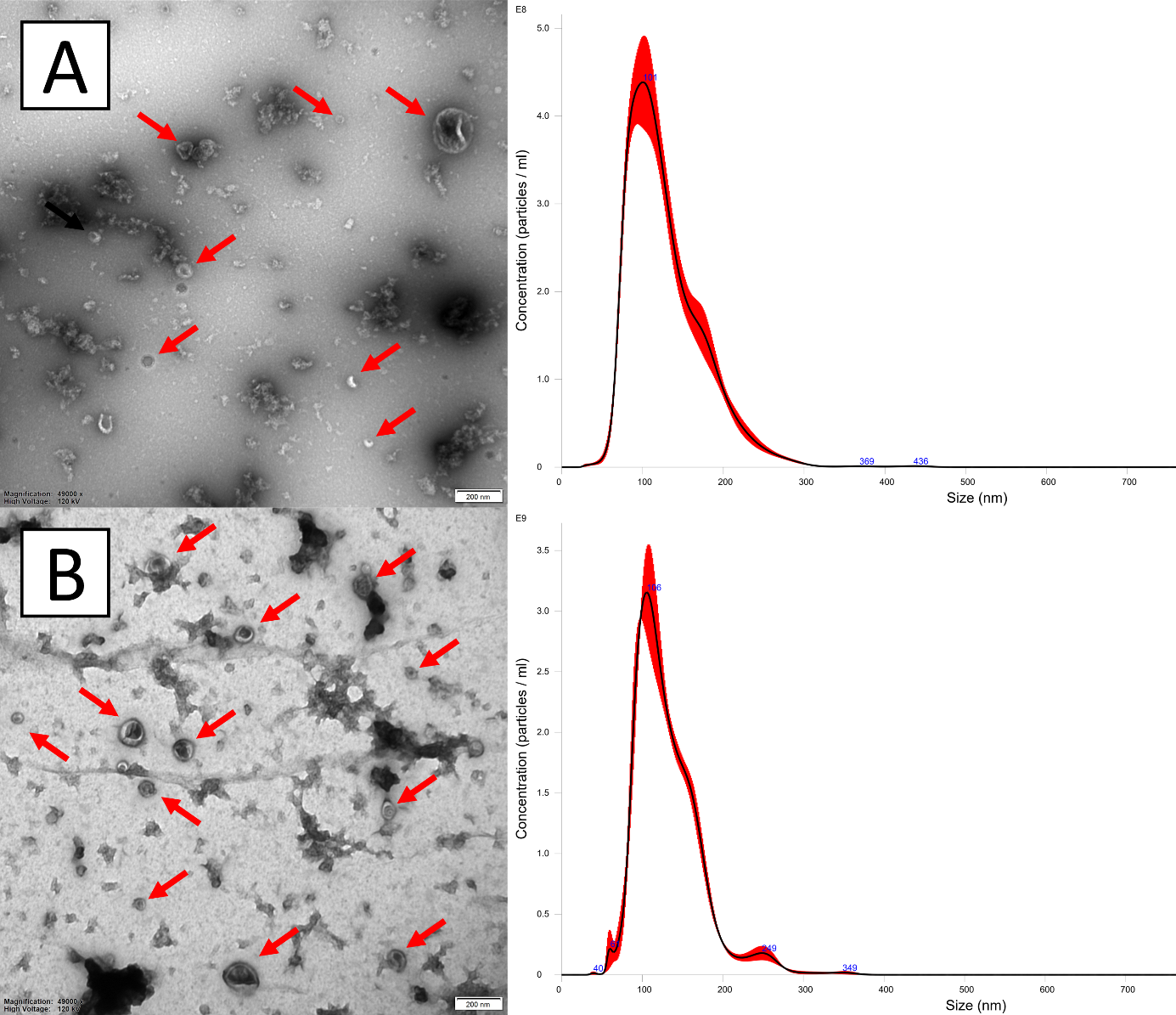
***

***Fig. S1*** *The exemplary NTA measurements and TEM photographs of the 8^th^ fraction isolated with SEC from CHO cells culturing media: (A) untransfected, (B) transfected by BChE expressing vector. Arrows indicate some examples of vesicles.*


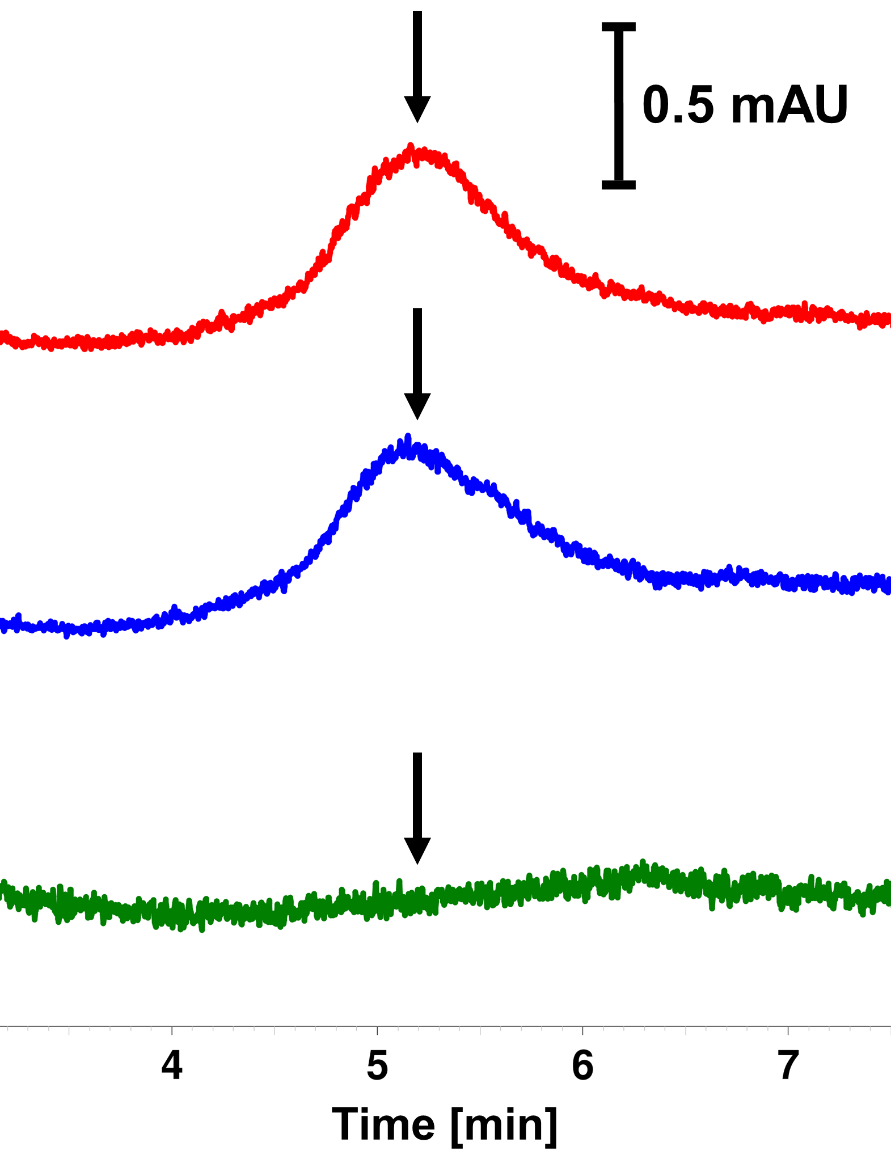


***Fig. S2*** *The CE analysis of the 8^th^ fractions, obtained with SEC from the culturing media of CHO cells (green) untransfected and transfected with vectors expressing genes encoding (blue) BChE, and (red) β-Gal. The arrows indicate the EVs peak.*
